# Changes in secondary metabolites in soybean (*Glycine max* L.) roots by salicylic acid treatment and their anti-LDL oxidation effects

**DOI:** 10.3389/fpls.2022.1000705

**Published:** 2022-09-26

**Authors:** Jeong Ho Kim, Abdul Bari Shah, Yong Hyun Lee, Aizhamal Baiseitova, Yeong Jun Ban, Ki Hun Park

**Affiliations:** Division of Applied Life Science (BK21 Plus), IALS, Gyeongsang National University, Jinju, South Korea

**Keywords:** soybean roots, coumestrol, daidzein, salicylic acid, anti-LDL oxidation

## Abstract

Abundance of metabolites in plant is a critical factor toward being functional food stuff. Salicylic acid (SA) treatment led significant changes in levels of the secondary metabolites in soybean roots. Notably, the exposure of 3 mM of SA aqueous solution to soybean plants for 24 h resulted in distinctive increases in the levels of coumestrol (16-fold, 0.3–4.8 mg/g DW) and daidzein (7-fold, 1.2–8.9 mg/g DW) in roots part. These changes were systematically investigated by LC-ESI-TOF/MS analysis to afford a clear difference of PLS-DA score, heatmap, and box plots. Quantitative analysis showed that SA treatment played to stimulate biosynthesis of coumestrol as well as hydrolysis of its glycosides (coumestrin and malonylcoumestrin). The highly improved anti-LDL oxidation effect was observed in the SA treated soybean roots in the three different assay systems. It might be rationalized by the increased levels of coumestrol and daidzein.

## Introduction

The health benefits of plant-based resources may be related to the presence and abundance of bioactive compounds in plants (Baenas et al., 2014). The abundance level of bioactive compounds can be increased with different tools such as molecular breeding (MB), genetically modified organisms (GMOs), and the application of elicitation tools. The MB and GMO approaches have practical limits in terms of the time they require to implement and their biosafety, respectively (Prakash et al., 2011).

A large number of studies have proven that elicitation seems to be a promising alternative to other conventional techniques for raising the levels of bioactive components in plants (Pérez-Tortosa et al., 2012; Anusuya and Sathiyabama, 2016; Wang et al., 2020). In fact, elicitors are widely used for increasing the level of secondary metabolites in plant cells, tissues, or the whole plant, and a diverse range of elicitors, such as phytohormones, polysaccharides, amino acids, and organic acids, have been used (Liu et al., 2019). Elicitations have been reported to enhance the level of phenolic compounds (Yamamoto et al., 2020), glucosinolates (Schreiner et al., 2011), vitamins (No et al., 2003), and γ-aminobutyric acid (Hao et al., 2016). However, few studies concerned with sufficiently enhancing the levels of bioactive compounds such that they are useful for nutraceutical purposes, have been reported.

Plant roots are located in the region close to the rhizosphere, where various interactions with microbial communities occur through phytochemicals (Bakker et al., 2013). In particular, soybean (*glycine max* L.) is a representative plant that interacts with microorganisms in the rhizosphere (Sugiyama, 2019). Soybean roots contain a series of isoflavones and pterocarpans with diverse biological benefits (Yuk et al., 2011; Ban et al., 2020). This study focused on increasing the bioactive metabolites in soy roots with the aid of salicylic acid (SA) belonging to plant hormones (Brunswick, 1992). SA is well known to elicit the production of secondary metabolites in response to many pathogens (Maruri-López et al., 2019; Ding and Ding, 2020). There is a report that SA influenced changes in phenolic metabolites of soybean seedlings (Durango et al., 2018).

Coumestrol is a phytoalexin in the soybean plant and is associated with insect attacks and senescence to which SA as a phytohormone connects (Morandi et al., 1984; Boué et al., 2000; Mun et al., 2021). In addition, coumestrol is considered as a valuable phytochemical with diverse biological activities such as its anti-low density lipoprotein (anti-LDL) oxidation (Jin et al., 2006), anti-inflammation (Yuk et al., 2017), anti-cancer (Zafar et al., 2017), anti-obesity (Kim et al., 2020b), and skin protection (Park et al., 2015) benefits. Daidzein is another important phytochemical that is highly sought after in the nutraceutical field owing to its antioxidant properties (Dwiecki et al., 2009), its use as a plant-based alternative to estrogen (Vitale et al., 2013), and its transformation potential to equal (Legette et al., 2014).

On the other hand, low density lipoprotein (LDL) is easily oxidized to oxLDL by reactive oxygen species (ROS; Chen et al., 2003). LDL oxidation prompts an early atherosclerosis process through several steps including endothelial cell damage, a form of cell accumulation, and the synthesis of autoantibodies (Pirillo, 2013; Mundi et al., 2018). Thus, substances with anti-LDL oxidative properties have been attractive natural sources of nutraceuticals. Pterocarpans, including coumestrol, were reported to display significant anti-LDL oxidation activities (Jin et al., 2006). These results, together with results from previous reports, suggest that coumestrol enriched soybean roots would be potentially useful because of their anti-LDL oxidation effects.

In this study, we observed the changes in the metabolites in soybean roots by treating the roots with SA to afford coumestrol and daidzein enriched soybean roots (CDESR). The detailed changes in the metabolites were systematically identified by LC-ESI-Q-TOF/MS and reported as the PLS-DA score, heatmap, and box plots. The anti-LDL oxidation effects of the CDESR were assessed with three different assay systems.

## Materials and methods

### Plant material and experimental design

Soybean [*G. max* (L.) Merrill] seeds were obtained from the National Institute of Crop Science (NICS), Miryang, Republic of Korea. The soybean plants were cultivated in pots in a greenhouse over a period of about 30 days until the plant reached the V3 growth stage (three nodes on the main stem with fully developed leaves). Experiments were conducted by exposing the plants to two different chemicals [SA and sodium salicylate (SS)]. After rinsing the roots with water, the plants were soaked in an aqueous solution of SA or SS at five concentrations: 1, 2, 3, 4, or 5 mM, following which the plants were placed in a chamber maintained at 25°C and a 16 h photoperiod for the duration of the treatment. The control plants were soaked in aqueous solution without SA and maintained for same time with treatment groups (Supplementary Figure S1). The plant roots were harvested 5 times (0, 3, 6, 12, and 24 after treatment) for time dependent effects. Samples for metabolomic analysis were collected randomly with 9 replications. The harvested samples were dried at a temperature of 35°C, milled to a powder in an electric mill and stored at-20°C for further experiments.

### HPLC-DAD analysis of phenolic metabolites

The milled soybean root samples (1.0 g) were extracted using 80% ethanol (50 ml) with sonication for 3 h at 30°C. After extraction, the samples were centrifuged at 5000 rpm for 15 min. The supernatants were filtered with a syringe filter (0.2 μm) and analyzed by HPLC. HPLC analysis of the extracts was conducted using an Agilent 1,200 series HPLC system including an Agilent G1311 A quaternary pump (Agilent Technologies, Santa Clara, CA) and Agilent G1315B diode array detector connected to a Zolbax Bonus-RP (150 mm × 4.6 mm, 5 μm) column (Agilent Technologies, Santa Clara, CA). Data were collected and analyzed with Chem Station software (Agilent Technologies). A linear gradient elution was carried out with water/0.1% acetic acid (A) and acetonitrile (B). The procedures for the gradient elution were as follows: 5 min, 85% A; 20 min, 80% A; 50 min, 50% A; 65 min, 40% A; 75 min, 0% A. The flow rate was 1.0 ml/min, 10 μl of each sample was injected into the column, and the absorbance was detected at 254 or 340 nm using the Agilent diode array detector. The phenolic compounds in the extracts were identified by comparison of the retention times (*t*_R_) of the compounds with the HPLC profiles of commercial standards and quantified by using their calibration curves obtained by determining the peak areas from the chromatograms (Supplementary Figure S2). The standards of daidzin, daidzein, genistin, genistein, and coumestrol were purchased from Sigma-Aldrich Co., Ltd. (St. Louis, MO, United States), and those of malonyldaidzin and malonylgenistin were purchased from MedChemExpress (NJ, United States).

### UPLC–Q-TOF/MS analysis

The metabolomic profiles in various extracts of soybean roots were analyzed on an ultra-performance liquid chromatography (UPLC) system manufactured by Waters Technologies (Waters, Milford, MA, United States). The metabolites were separated in an Acquity UPLC BEH C 18 column (2.1 × 100 mm, 1.7 μm, Waters) with a flow rate of 0.35 ml/min at 30°C. The mass spectrometer was operated in positive electrospray ionization (ESI) mode and the TOF-MS and MS/MS results of the metabolites were collected in the *m*/*z* 50–1,500 range with a scan time of 0.2 s. The other mass spectrometric conditions were configured as follows: temperature of source, 100°C; desolvation, 400°C; sampling cones voltage, 30 V; capillary voltage, 3 kV; flow rate of desolvation, 800 l/h; collision energy, 10 to 30 eV. The mobile phase was comprised of solvent A (0.1% formic acid in water) and solvent B (0.1% formic acid in acetonitrile) using a gradient elution of 0–1 min, 3% B; 1–5 min, 3−15% B; 5–10 min, 15−25% B; 10–12 min, 25−45% B; and 12–20 min, 45–100% B. For quality control (QC), a mixture of all samples was injected after every 6 samples. The MS data including the retention time (*t_R_*) and *m*/*z* were obtained using MassLynx software (Waters) and the ion intensities were acquired by MarkerLynx software (Waters).

### Data processing and statistical analysis

The data sets from the UPLC-ESI-Q-TOF/MS analysis were collected, aligned, and normalized by MarkerLynx software (Waters). The peak of each metabolite was collected as follows: peak width at 5% height of 0.5 s, and peak-to-peak baseline noise of 1,000. The respective metabolites were identified based on Chemspider,^1^ the METLIN database (metlin.scripps.edu), and human metabolome databases.^2^ The aligned and normalized LC/MS data sets were multivariately analyzed with SIMCA-P^+^ version 16.0.2 (Umetrics, Umeå, Sweden). Partial least squares discriminant analysis (PLS-DA) was carried out to visualize discrimination between the control group and the groups exposed to SA. Hotelling’s T2 test was employed to exclude outliers from the 95% confidence region. The quality of the PLS-DA model was evaluated using the following three parameters: *R*^2^*X*, *R*^2^*Y*, and *Q*^2^*Y*. The improvement of fit was quantified by *R*^2^*X* and *R*^2^*Y* and the predictive ability was determined by *Q*^2^*Y*. The PLS-DA models were validated using 7-fold cross validation and the reliability was evaluated by a permutation test (*n* = 200). The metabolites contributing to the discrimination in the sample groups were found and then determined by variable significance in the projection (VIP) value >0.7, intended by PLS-DA with one-way analysis of variance (ANOVA) with Duncan’s test (*p* < 0.05) using SPSS 17.0 (SPSS Inc., United States). The selected metabolites with significant differences (*p* < 0.05) were also visualized in a heat map described using *R* with gplots.

### Measurements of thiobarbituric acid reactive substances

The thiobarbituric acid reactive substances (TBARS) assay was conducted according to an existing method with minor alteration (Jin et al., 2006). The absorbance of the product of the reaction between malondialdehyde (MDA) and thiobarbituric acid (TBA) was measured at 532 nm. Briefly, 10 μl of 3 mg/ml of LDL (ProSpec, Rehovot, Israel) solution in 220 μl of PBS buffer (10 mM, pH 7.4), 10 μl of 0.25 mM CuSO_4_, and 10 μl of different concentrations of compounds and soybean root extracts or probucol as a positive control were mixed in a 1.5 ml Eppendorf tube and incubated at 37°C for 24 h. After incubation, 100 μl of trichloroacetic acid (20% w/v) and 100 μl of 0.67% thiobarbituric acid (0.67% w/v dissolved in 0.05 N NaOH) were added to the mixture and heated in the water bath at 100°C for 30 min. After heating, the mixture was cooled on ice and centrifuged for 10 min at 5000 rpm. The absorbance of the supernatant was determined and the IC_50_ values of each sample were calculated using the inhibition rate obtained by Equation (1).


(1) Inhibition rate%=100×rate of control−rate of inhibitor/rate of control 

### Measurements of conjugated diene formation

The formation of conjugated diene (CDs) from oxLDL was measured using a previously reported method (Xu et al., 2007). First, 1 ml of 50 μg/ml of LDL solution in PBS buffer (10 mM, pH 7.4) was incubated with different concentrations of compounds, soybean root extract, or probucol as a positive control. The control experiments were carried out under equivalent assay conditions but without adding the above compounds or extracts. The oxidation commenced immediately after adding CuSO_4_ such that the final concentration was 10 μM. The formation of CDs was monitored at 234 nm for 240 min in increments of 5 min, continuously, using a SpectraMax^®^ M3 multi-mode microplate reader (Molecular Devices, CA, United States). On the acquired absorbance spectra, the lag time of each sample was observed as the endpoint indicated in the lag phase.

### Relative electrophoretic mobility

The relative electrophoretic mobility (REM) of native LDL and oxidized LDL induced by Cu^2+^ was measured according to procedures in previous reports (Kim et al., 2020a). Before electrophoresis, the reaction mixtures including the 10 μM CuSO_4_ and the compounds, samples of soybean root extract, or probucol as a positive control at various concentrations were added to the LDL solution (500 μg/ml), then incubated at 37°C for 16 h in the dark. The native LDL and premixed LDL samples were loaded onto 0.5% agarose gel in TAE buffer (40 mM Tris-acetate with 1 mM EDTA, pH 8.0) and electrophoresed for 50 min at 100 V. After the electrophoresis, the gel was fixed in 40% ethanol with 10% acetic acid for 30 min, stained with 0.15% coomassie brilliant blue R250, and the LDL bands were visualized by a destaining solution (methanol:acetic acid:water, 3:2:15, v/v).

## Results and discussion

### Effects of the application of salicylic acid to soybean roots

**Figure 1:**
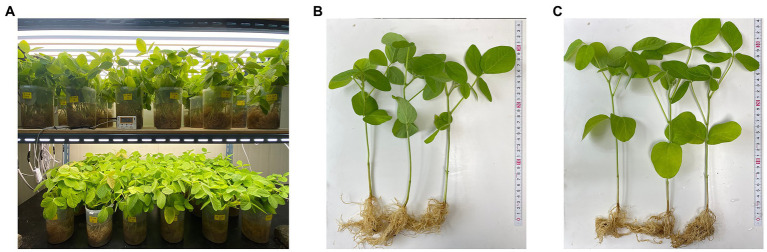
**(A)** Process of salicylic acid (SA) treatment on soybean plants. **(B)** Control soybean plants. **(C)** Soybean plants after treatment with 3 mM of SA for 24 h.

Soybean plants secrete unique metabolites from their roots to maintain a symbiotic relationship with rhizobia (Han et al., 2020) and mycorrhiza fungi (Pawlowski et al., 2020). The major metabolites in the roots are mostly isoflavones and their derivatives, all of which reportedly are beneficial for human health (Wang et al., 2013). In this study, soybean roots were observed to undergo a distinctive ingredient change upon exposure to SA. We applied SA aqueous solutions ranging 1 to 5 mM at the V3 growth stage (30 days after sowing). The level of metabolites were changed according to SA concentrations and exposure time (Supplementary Figures S3–S5). The optimal condition were observed 3 mM of SA solution and 24 h of exposure time. The visible appearance of the soybean plant did not differ much during its exposure to SA under these conditions as shown in Figure 1; Supplementary Figure S1.

**Figure 2:**
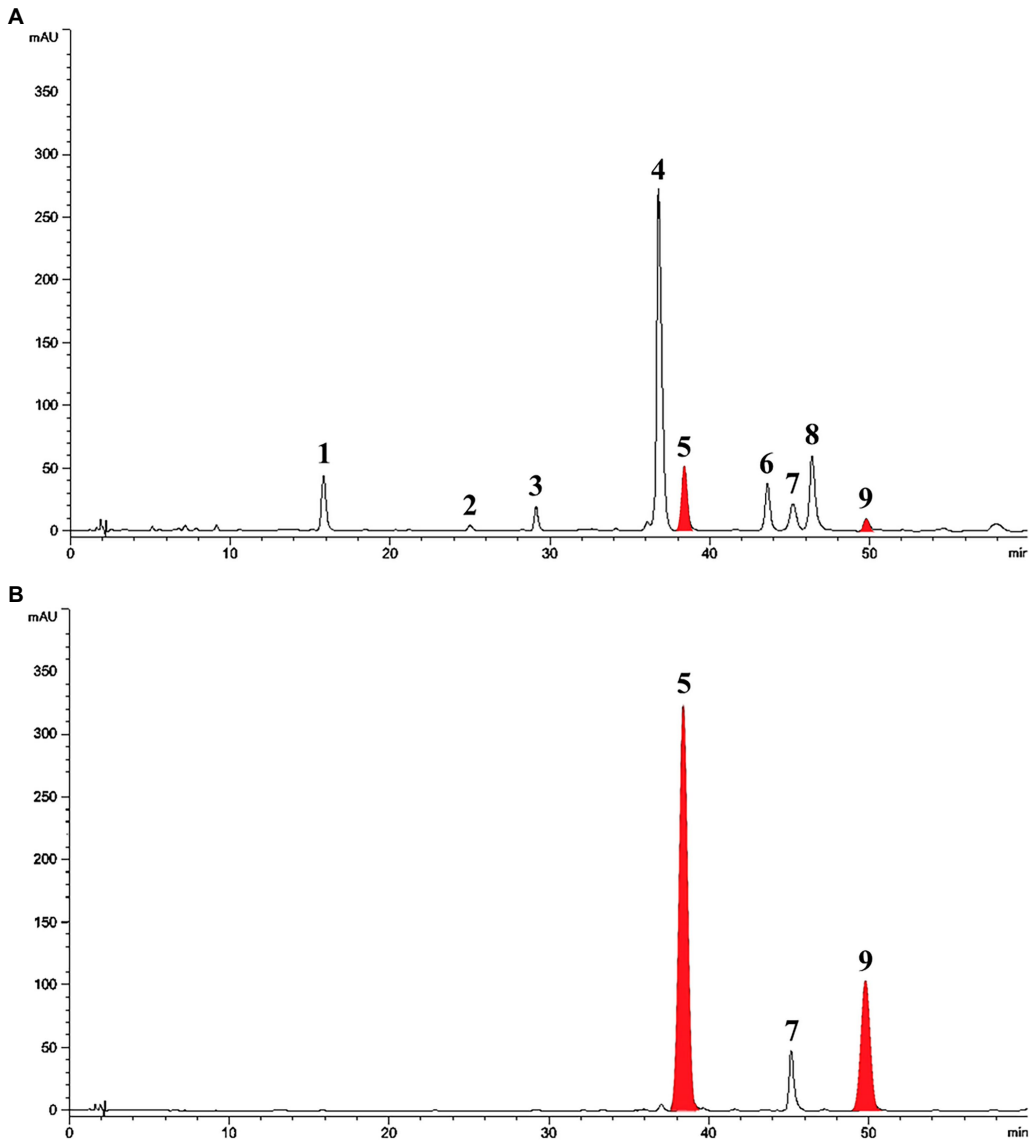
HPLC chromatographic patterns of soybean root extracts at 254 nm. Peak 1, daidzin; peak 2, genistin; peak 3, coumestrin; peak 4, malonyldaidzin; peak 5, daidzein; peak 6, malonylgenistin; peak 7, genistein; peak 8, malonylcoumestrin; peak 9, coumestrol. **(A)** Control soybean roots. **(B)** SA treated soybean roots.

**Figure 3:**
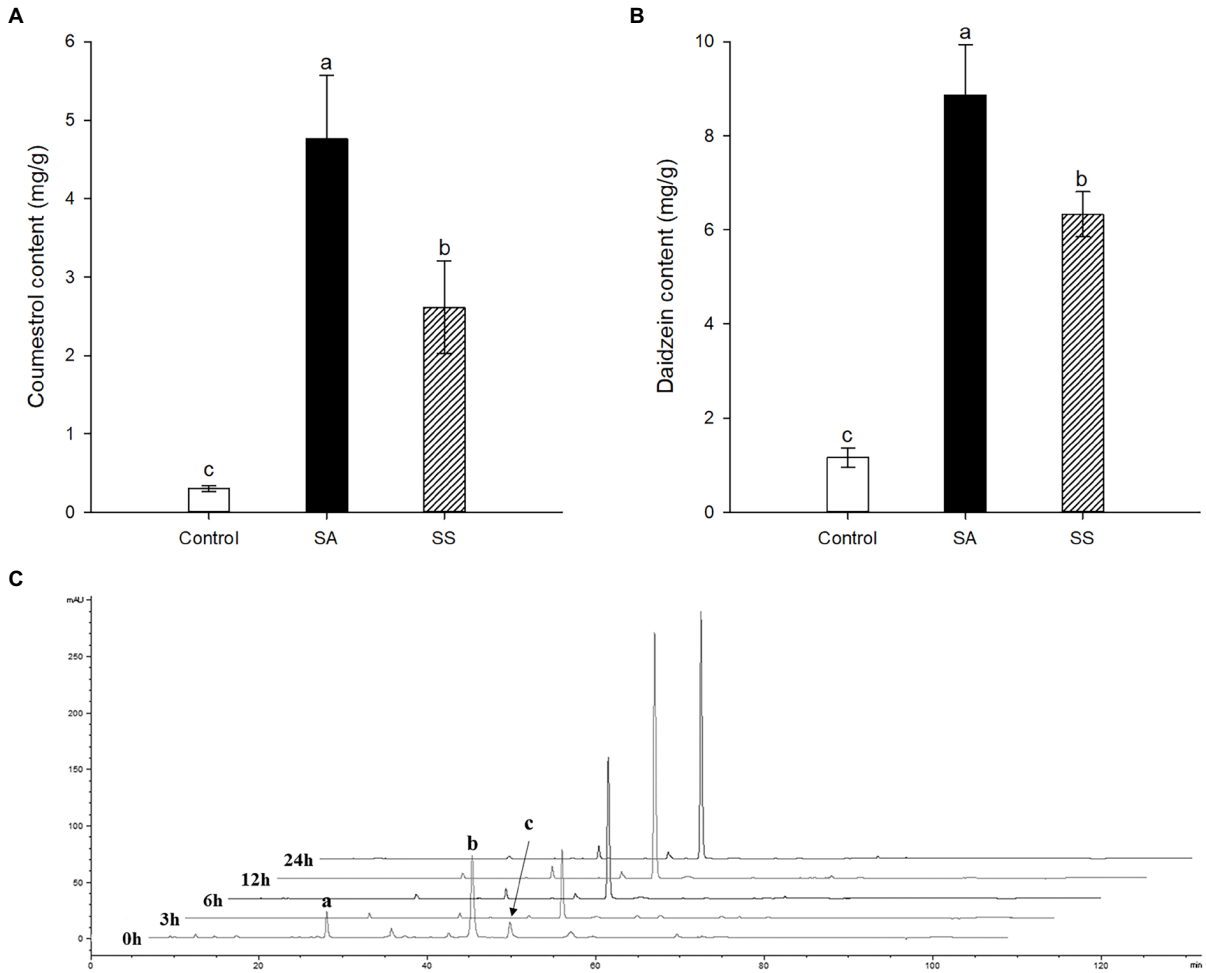
**(A)** Comparison of coumestrol contents between SA and sodium salicylate (SS) treatments. **(B)** Comparison of daidzein contents between SA and SS treatments. **(C)** Changes in HPLC chromatographic patterns by treatment time of SA at 340 nm. Peak a, coumestrin; peak b, malonylcoumestrin; peak c, coumestrol. Data are the mean ± SD of determinations performed in five replicates in 5 samples. Different letters indicate significant differences, as determined by Tukey’s (LSD) test with *p* < 0.05.

Treatment with SA afforded a dramatic increase in the levels of daidzein and coumestrol in the roots, as was evident from the red band on the HPLC chromatogram (Figures 2A,B) and the results of the quantitative analysis (Table 1). Their specific changes are discussed in the section on metabolomic analysis (*vide infra*). We assumed that daidzein (peak 5) and coumestrol (peak 9) emerged as a result of the hydrolysis of their corresponding glycosides, namely daidzin (peak 1), malonyldaidzin (peak 4), coumestrin (peak 3), and malonylcoumestrin (peak 8). In particular, the content of coumestrol was increased 16-fold to be 4.76 mg/g DW from 0.30 mg/g DW. Enzymatic hydrolysis experiments indicated that the contents of coumestrin (peak 3) and malonylcoumestrin (peak 8) were equivalent to 2.14 mg/g DW of coumestrol as shown in Supplementary Figure S6. This may be explained by the activation of β-glucosidase in the chemical defense process of the plant by signal molecules including SA (Morant et al., 2008). The SA application also stimulated production of coumestrol which was *ca.* 55% of total amount (2.62 mg/g DW). Meanwhile, SS also effectively enhanced the daidzein and coumestrol content, but the induction yields were 55% and 71%, respectively, compared with the yields after SA treatment (Figures 3A,B). In the presence of SA, the soybean roots started accumulating coumestrol and daidzein, the concentrations of which increased rapidly and continuously for 24 h as shown in Supplementary Figures S4, S5. Figure 3C shows the dependence of the coumestrol content on the exposure time to SA: 0 h (0.30 mg/g DW), 3 h (1.35 mg/g DW), 6 h (2.75 mg/g DW), 12 h (4.73 mg/g DW), and 24 h (4.76 mg/g DW). Quantification of phytoestrogens in Legumes (Franke et al., 1994) indicated that most of plants possess a trace amount of coumestrol. The most coumestrol abundant plant sources are clover sprout (5.6 mg/g DW) and alfalfa sprout (0.7 mg/g DW), but they have no daidzein. When we consider the levels of daidzein and coumestrol together, the CDESR might be the plant source with the highest level of coumestrol and daidzein.

**Table 1:** Contents (mg/g DW) of dietary phytoestrogens in soybean roots.

Compound	Control^a^	CDESR^b^
Daidzin	0.92^c^ ± 0.22	ND^d^
Malonyldaidzin	14.77 ± 2.55	ND
Daidzein	1.16 ± 0.2	8.87 ± 1.06
Genistin	0.12 ± 0.01	ND
Malonylgenistin	1.86 ± 0.15	ND
Genistein	0.11 ± 0.03	0.88 ± 0.07
Coumestrin	NA^d^	ND
Malonylcoumestrin	NA	ND
Coumestrol	0.30 ± 0.04	4.76 ± 0.81

a Not treated.

b Coumestrol and daidzein enriched soybean roots treated with 3 mM of salicylic acid for 24 h.

c All values are mean ± SD of in six independent experiments; Not detected.

d Not commercially available.

SA is well known to be induced in plants in response to many pathogens and then this elicits the production of secondary metabolites (Ding and Ding, 2020). Many case studies of the application of SA have been conducted with the aim of enhancing the production of stilbenes (Xu et al., 2015), alkaloids (Figueroa-Pérez et al., 2015), anthraquinones (Lee et al., 2013), terpenoids (Wen Xu, 2012), etc. Our results would be a comparative case study regarding SA application to introduce secondary metabolites in plants.

### Metabolomic analysis of soybean roots with UPLC-ESI-Q-TOF/MS

**Figure 4:**
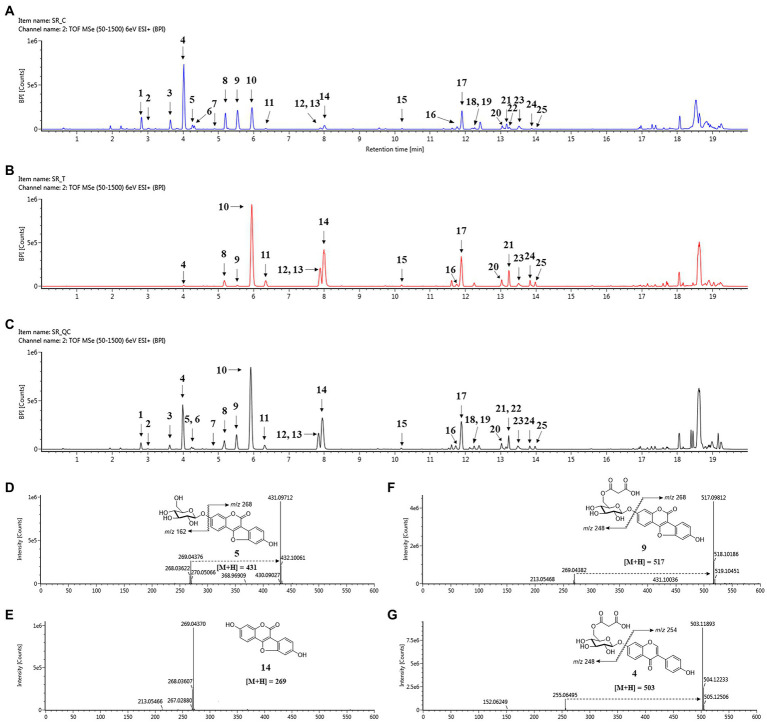
Changes in secondary metabolites of soybean roots by SA treatment. **(A)** BPI chromatogram of control soybean roots. **(B)** BPI chromatogram of SA treated soybean roots. **(C)** BPI chromatogram of quality control (QC). **(D–G)** Positive ion mass spectra of representative metabolites of soybean roots: **(D)** Coumestrin, **(E)** Malonylcoumestrin, **(F)** Coumestrol, **(G)** malonyldaidzin. Peak 1, daidzin; peak 2, glycitin; peak 3, genistin; peak 4, malonyldaidzin; peak 5, coumestrin; peak 6, malonylglycitin; peak 7, acetyldaidzin; peak 8, malonylgenistin; peak 9, malonylcoumestrin; peak 10, daidzein; peak 11, glycitein; peak 12, isotrifoliol; peak 13, genistein; peak 14, coumestrol; peak 15, soyasaponin Ab; peak 16, glyceollin I; peak 17, soyasaponin I; peak 18, soyasaponin II; peak 19, soyasaponin III; peak 20, soyasaponin αg; peak 21, soyasaponin βg; peak 22, phaseol; peak 23, soyasaponin βα; peak 24, γg; peak 25, glyceollin IV.

The metabolomic analysis was designed to determine the changes in the metabolites in soybean roots resulting from treatment with SA. In this regard, 80% ethanol was found to be appropriate solvent for maximal and well-balanced extraction in view of diverse polarities of metabolites. An analysis of the profiles of metabolites using UPLC-ESI-Q-TOF/MS in positive ion mode led to the detection of a total of 176 metabolites in soybean roots. A comparison of the representative base peak intensity (BPI) chromatogram within 20 min showed that the metabolites in the roots were of a similar type, but the levels of many metabolites differed as shown in Figures 4A–G. The system stability and quality of the obtained data were evaluated to be reliable by correlating the QC samples (Figure 4C). The PLS-DA score plots were prepared from the metabolites with VIP > 0.7 among the sample groups which contributed to separation (Supplementary Table S1).

The 25 major metabolites were annotated by using their molecular weight ([M + H]^+^), the major fragment of their TOF/MS spectral data, and a comparison with previous reports (Table 2; Jin et al., 2006; Yuk et al., 2011; Ban et al., 2020). First, ten isoflavone peaks were annotated by identical molecular ion [M + H]^+^ at *m*/*z* 417.1176 (peak 1, daidzin), *m*/*z* 447.1284 (peak 2, glycitin), *m*/*z* 433.1128 (peak 3, genistin), *m*/*z* 503.1189 (peak 4, malonyldaidzin), *m*/*z* 533.1296 (peak 6, malonylglycitin), *m*/*z* 459.1288 (peak 7, acetyldaidzin), *m*/*z* 519.1136 (peak 8, malonylgenistin), *m*/*z* 255.0646 (peak 10, daidzein), *m*/*z* 285.0753 (peak 11, glycitein), and *m*/*z* 271.0595 (peak 13, genistein). The seven pterocarpans were confirmed by identical molecular ion [M + H]^+^ at *m*/*z* 431.0971 (peak 5, coumestrin), *m*/*z* 517.0981 (peak 9, malonylcoumestrin), *m*/*z* 299.0543 (peak 12, isotrifoliol), *m*/*z* 269.0437 (peak 14, coumestrol), *m*/*z* 339.1220 (peak 16, glyceollin I), *m*/*z* 337.1064 (peak 22, phaseol), and *m*/*z* 355.1534 (peak 25, glyceollin IV). The eight soyasaponin peaks were annotated by identical molecular ion [M + H]^+^ at *m*/*z* 1437.6557 (peak 15, soyasaponin Ab), *m*/*z* 943.5263 (peak 17, soyasaponin I), *m*/*z* 797.4693 (peak 18, soyasaponin III), *m*/*z* 913.5161 (peak 19, soyasaponin II), *m*/*z* 1085.5523 (peak 20, soyasaponin αg), *m*/*z* 1069.5565 (peak 21, soyasaponin βg), *m*/*z* 1039.5466 (peak 23, soyasaponin βα), and 923.5010 (peak 24, soyasaponin γg). The specific MS data are summarized in Table 2; Supplementary Figure S7.

**Table 2:** Peak assignments of secondary metabolites in soybean roots by UPLC–ESI-TOF/MS.

Peak	*t_R_* (min)	Elemental composition	Molecular ion [*M* + *H*]^+^ (*m*/*z*)	Observed fragment ions [*M* + *H*]^+^ (*m*/*z*)	Idendification
1	2.82	C_21_H_20_O_9_	417.1176	255.0647	Daidzin
2	3.08	C_22_H_22_O_10_	447.1284	285.0756	Glycitin
3	3.64	C_21_H_20_O_10_	433.1128	271.0595	Genistin
4	4.02	C_24_H_22_O_12_	503.1189	255.0649	Malonyldaidzin
5	4.27	C_21_H_18_O_10_	431.0971	269.0437	Coumestrin
6	4.31	C_25_H_24_O_13_	533.1296	285.0757	Malonylglycitin
7	4.91	C_23_H_22_O_10_	459.1288	255.0653	Acetyldaidzin
8	5.20	C_24_H_22_O_13_	519.1136	271.0596	Malonylgenistin
9	5.54	C_25_H_24_O_12_	517.0981	269.0438	Malonylcoumestrin
10	5.95	C_15_H_10_O_4_	255.0646		Daidzein
11	6.34	C_16_H_12_O_5_	285.0753		Glycitein
12	7.86	C_16_H_10_O_6_	299.0543	271.0603, 255.0284	Isotrifoliol
13	7.89	C_15_H_10_O_5_	271.0595		Genistein
14	8.00	C_15_H_8_O_5_	269.0437		Coumestrol
15	10.20	C_67_H_104_O_33_	1437.6557	975.5156, 439.3580, 169.0488	Soyasaponin Ab
16	11.76	C_20_H_18_O_5_	339.1220	321.1113, 305.0811	Glyceollin I
17	11.90	C_48_H_78_O_18_	943.5263	423.3620	Soyasaponin I
18	12.26	C_42_H_68_O_14_	797.4693	423.3621	Soyasaponin III
19	12.26	C_47_H_76_O_17_	913.5161	617.4060, 441.3727, 423.3621	Soyasaponin II
20	13.08	C_54_H_84_O_22_	1085.5523	571.2404, 543.2803, 423.3619	Soyasaponin αg
21	13.17	C_54_H_84_O_21_	1069.5565	563.2436, 423.3619	Soyasaponin βg
22	13.24	C_20_H_16_O_5_	337.1064	281.0441, 253.0503	Phaseol
23	13.46	C_53_H_82_O_20_	1039.5466	743.4381, 423.3616	Soyasaponin βα
24	13.76	C_48_H_74_O_17_	923.5010	423.3623	Soyasaponin γg
25	13.88	C_21_H_22_O_5_	355.1534	337.1428, 335.1276, 245.1172	Glyceollin IV

**Figure 5:**
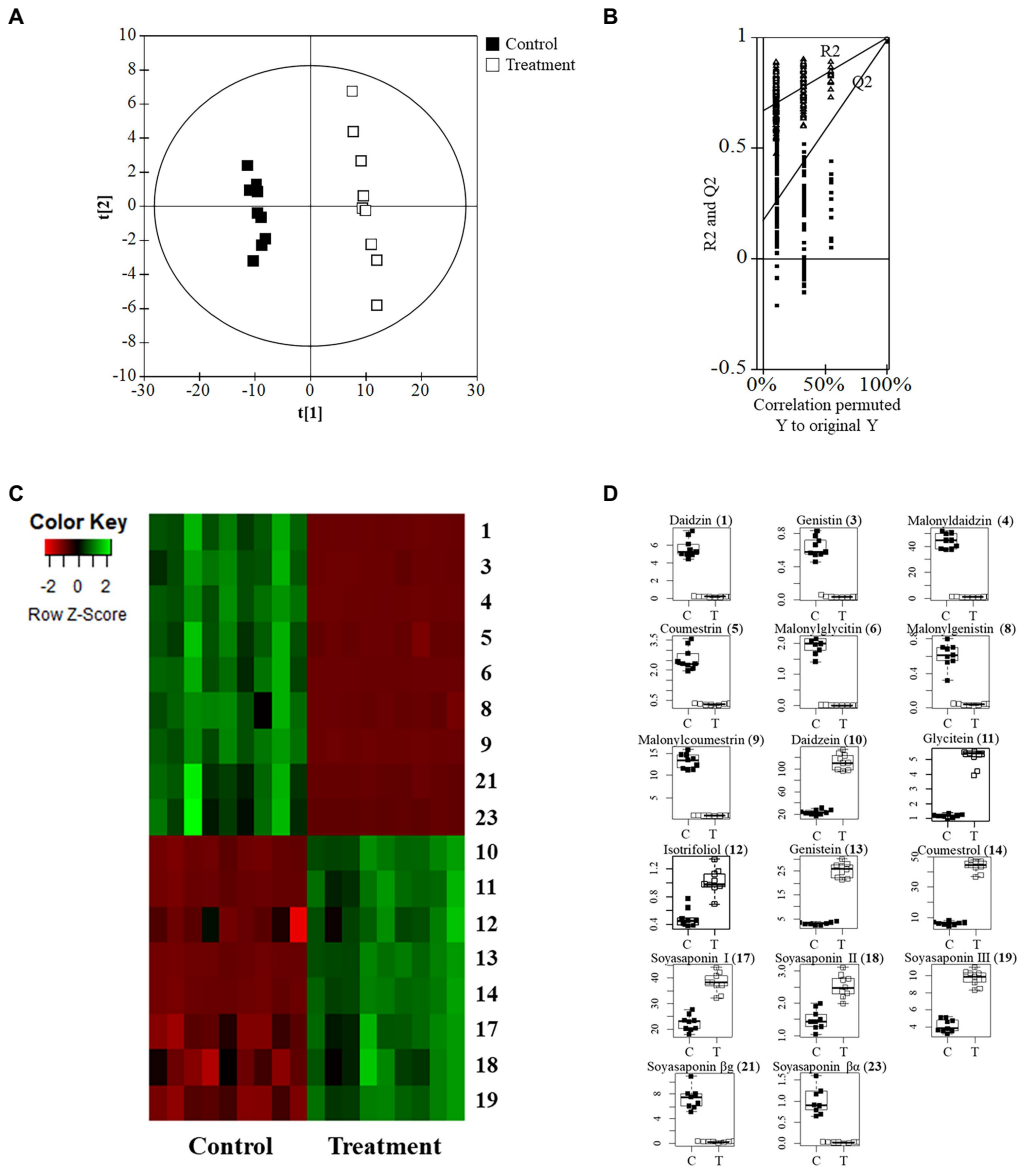
Metabolomic analysis of soybean roots by LC-TOF/MS. **(A)** PLS-DA plots of untreated (control) and SA treated roots. C, control group; T, treatment group. **(B)** Performance of the permutation tests validated from the PLS-DA model. **(C)** Heatmap for the 17 metabolites (*p* < 0.05) between control and treatment. **(D)** Boxplots of representative metabolites.

The quality parameters of the model used for the PLS-DA analysis were calculated as having the following values: *R*^2^*X* = 0.648, *R*^2^*Y* = 0.995, and *Q*^2^ = 0.983. The permutation validation was reliable and verified the PLS-DA analysis: *R*^2^ intercept = 0.673, *Q*^2^ intercept = 0.169, and *value of p* < 0.05. The groups of metabolites in the soybean roots were significantly different on the two-component PLS-DA score plots (Figures 5A,B). The profiling overview of these different metabolites in the pairwise comparison of the two groups is shown in the form of a heatmap (Figure 5C). The control group and that subjected to SA treatment were clearly separated into two clusters as indicated by the green-red color scale, which was obtained from the *z*-score transformed raw data of the metabolites. The 17 metabolites were selected to be significantly affected by exposure to SA based on higher VIP values than 1.0. The 17 different metabolites were assigned to the flavonoid and soyasaponin categories. The metabolites with increased levels were annotated, namely daidzein (**10**), glycitein (**11**), isotrifoliol (**12**), genistein (**13**), coumestrol (**14**), soyasaponin I (**17**), soyasaponin III (**18**), and soyasaponin II (**19**). The decreased metabolites were daidzin (**1**), genistin (**3**), malonyldaidzin (**4**), coumestrin (**5**), malonylglycitin (**6**), malonylgenistin (**8**), malonylcoumestrin (**9**), soyasaponin βg (**21**), and soyasaponin βα (**23**). The box plots in Figure 5D show that daidzein and coumestrol predominantly accumulated in the course of the SA treatment and that the levels of their corresponding glycosides in the soybean roots were significantly lower as a result. The levels of the soyasaponin metabolites were also affected in that those of three of them (**17**, **18**, and **19**) increased and two of them (**21** and **23**) decreased. The metabolomic analysis revealed that treatment with SA promoted significant changes in the levels of metabolites in soybean roots towards enhancing the value of these roots for nutraceutical purposes.

### Anti-LDL oxidation effects of soybean roots

In a previous study (Jin et al., 2006), coumestrol showed significant anti-LDL oxidation activity in four different assay systems, namely the TBARS, lag time of CD formation, relative electrophoretic mobility (REM) experiment, and fragmentation pattern of ApoB-100 protein. Daidzein also showed moderate inhibition against LDL oxidation. Coumestrol and daidzein enriched soybean roots (CDESR) were obtained by exposing the roots to SA. The anti-LDL oxidation effects of an 80% ethanol extract of CDESR were examined with TBARS, the lag time of CD formation, and REM experiments. All experiments were carried out with human LDL in the presence of 10 μM Cu^2+^ as an oxidation initiator.

**Figure 6:**
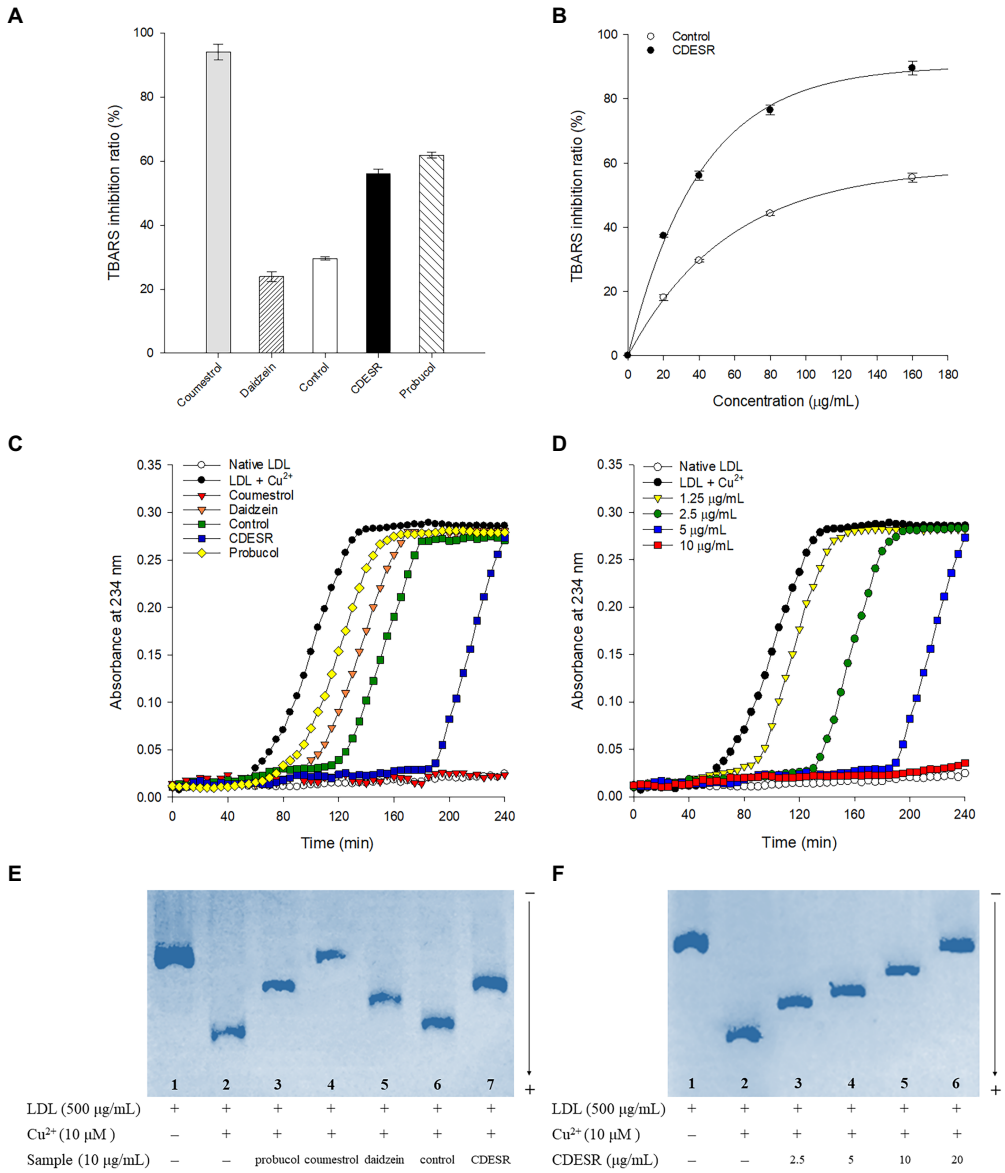
**(A)** Inhibitions of Cu^2+^-induced lipid peroxidation by 40 μg/ml of coumestrol, daidzein, control soybean roots extract, and CDESR (coumestrol and daidzein enriched soybean roots) extract in the TBARS assay. Probucol was used as a positive control. Data represent the mean ± SE (*n* = 3). **(B)** Dose-dependent effects of control roots and CDESR in the TBARS assay. Data represent the mean ± SE (*n* = 3). **(C)** Inhibitory effects (extension of lag time) of 5 μg/ml of samples on conjugated diene generation in Cu^2+^-induced LDL oxidation. **(D)** Dose-dependent effects of CDESR on conjugated diene generation. **(E)** Inhibitory effects on Cu^2+^ mediated LDL oxidation by relative electrophoretic mobility (REM). Lane 1, native LDL (absence of Cu^2+^); lane 2, oxidized LDL; lane 3–7, 10 μg/ml of probucol, coumestrol, daidzein, control root extract, and CDESR extract. **(F)** Dose-dependent effects of CDESR by REM assay. Lane 1, native LDL; lane 2, oxidized LDL; lane 3–6, CDESR (2.5 μg/ml– 0 μg/ml).

First, the anti-LDL oxidation effects of coumestrol, daidzein, control roots, and CDESR were assessed with a TBARS assay using a concentration of 40 μg/ml (Figure 6A). The CDESR blocked LDL-oxidation efficiently compared with the control roots and daidzein. The CDESR (IC_50_ = 36.1 μg/ml) were 3-fold more effective than the control roots (IC_50_ = 108.9 μg/ml) as shown in Supplementary Table S2. As reported previously (Jin et al., 2006), coumestrol completely inhibited LDL-oxidation at 40 μg/ml. The difference between CDESR and the control roots were doubly confirmed by the dose-dependence curve (Figure 6B).

The anti-LDL oxidation effects of all the entries were additionally assessed by examining their CD formation at 5 μg/ml for 240 min (Figure 6C). The anti-LDL oxidation potencies were demonstrated by determining the lag time in the process of incubation with 10 μM CuSO_4_. The control oxLDL recorded a lag time of 50 min, whereas probucol as a positive control extended the lag time to 60 min. Similar to the results of the TBARS assay, the anti-LDL oxidation of CDESR was more efficient in terms of its lag time (190 min), exceeding that of the control (115 min). The lag time became longer in proportion to the concentrations of CDESR (1.25–10 μg/ml) dose-dependently, as shown in Figure 6D.

In a third assessment of the anti-LDL oxidation properties, REM experiments were carried out to examine additional parameters. As shown in Figure 6E, oxLDL moved to the bottom because of its negative charge. The lanes (1–7) in Figure 6E were designed as follows: lane 1 (native LDL), lane 2 (oxidized LDL), and lane 3–7 (samples at 10 μg/ml), and all samples were incubated for 16 h. The mobility of LDL was clearly reduced by coumestrol, daidzein, and probucol as in the previous study (Jin et al., 2006). The efficacy of the CDESR inhibited LDL-oxidation was 78.2% compared with that of the control roots of 17.3% at 10 μg/ml. Dose-dependent inhibitions of CDESR were observed in REM experiments of 4ss0.8% (2.5 μg/ml), 56.3% (5 μg/ml), 78.2% (10 μg/ml), and 95.8% (20 μg/ml) as shown in Figure 6F; Supplementary Figure S8. The improved antioxidant potential of CDESR could be attributed to the dramatic increase in the coumestrol (0.3–4.76 mg/g DW) and daidzein (1.16–8.87 mg/g DW) levels as a consequence of the application of SA.

## Conclusion

In summary, the application of SA led to significant changes in the levels of secondary metabolites in soybean roots. Coumestrol and daidzein were revealed to be the metabolites of which the concentrations changed the most to reach levels as high as 4.76 mg/g DW and 8.87 mg/g DW, respectively. Metabolomic analyses were carried out by UPLC-ESI-Q-TOF/MS to reveal details of the changes in the metabolites by using the PLS-DA score, heatmap, and box plots. In particular, SA treatment played to stimulate a production of coumestrol as well as hydrolysis of its glycosides (coumestrin and malonylcoumestrin) in soybean roots. The SA treated soybean roots, CDESR, showed much improved anti-LDL oxidation effects than control roots based on the TBARS, CD formation, and REM experiments. This was rationalized by the distinctive increase in the coumestrol and daidzein levels in soybean roots as a result of SA application.

## Data availability statement

The original contributions presented in the study are included in the article/Supplementary material, further inquiries can be directed to the corresponding author.

## Author contributions

JK, YL, YB, and KP conceived and designed the experiments. AB and YB analyzed the data. JK wrote the manuscript. JK, AS, AB, YB, and KP were involved in the related discussion. AB and AS helped to improve the quality of the manuscript. All authors contributed to the article and approved the submitted version.

## Funding

This research was supported by the Bio and Medical Technology Development Program of the National Research Foundation (NRF) funded by the Ministry of Science and ICT (No. NRF2020M3A9I3038523) and Rural Development Administration (PJ015732), Republic of Korea. The BK21 plus program supported scholarships for all students. The researchers received no external funding.

## Conflict of interest

The authors declare that the research was conducted in the absence of any commercial or financial relationships that could be construed as a potential conflict of interest.

## Publisher’s note

All claims expressed in this article are solely those of the authors and do not necessarily represent those of their affiliated organizations, or those of the publisher, the editors and the reviewers. Any product that may be evaluated in this article, or claim that may be made by its manufacturer, is not guaranteed or endorsed by the publisher.

## Supplementary material

The Supplementary material for this article can be found online at: https://www.frontiersin.org/articles/10.3389/fpls.2022.1000705/full#supplementary-material

## Footnotes

^1^ www.chemspider.com

^2^ www.hmdb.ca
