# Supplementary material for: Changes in secondary metabolites in soybean (Glycine max L.) roots by salicylic acid treatment and their anti-LDL oxidation effects
Source: Front Plant Sci. 2022 Sep 26;13:1000705. doi: 10.3389/fpls.2022.1000705 (PMC9549270; doi:10.3389/fpls.2022.1000705)
Supplement: Supplementary file 1 [file Data_Sheet_1.docx]

**Supplementary material**

**Changes in secondary metabolites in soybean (*Glycine max* L.) roots by salicylic acid treatment and their anti-LDL oxidation effects**

**▶Characterization Data**

- Fig. S1 : Appearance of soybean plants during cultivation and salicylates treatment.

- Fig. S2 : Calibration curves of isoflavones and coumestrol standards by HPLC anlaysis.

- Fig. S3 : The changes in coumestrol and daidzein levels at different concentrations of SA.

- Fig. S4 : The changes in HPLC chromatogram patterns at 254 nm UV during SA application.

- Fig. S5 : The changes in HPLC chromatogram patterns at 340 nm UV during SA application.

- Fig. S6 : Enzymatic hydrolysis of soybean roots extracts.

- Fig. S7 : Mass fragmentation patterns of each metabolite in soybean roots.

- Fig. S8 : Dose-dependent effects of control soybean roots and CDESR in REM assay.

**-** Table S1 : Identification of the metabolites contributing the separation among sample groups.

**-** Table S2 : Effects of each compound or extract on oxidized LDL induced by Cu^2+^.


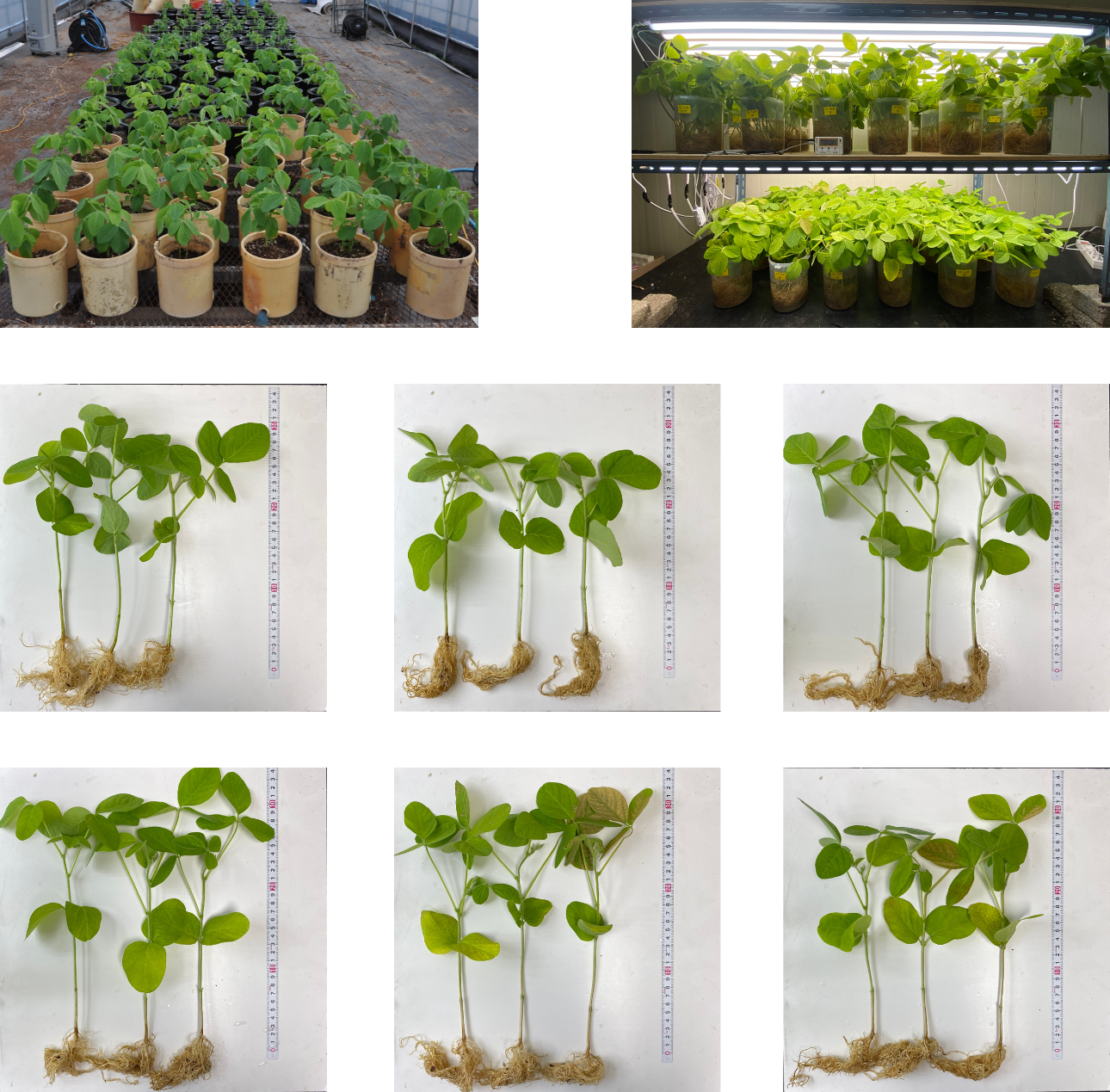
(A) (B)

(C) (D) (E)

(F) (G) (H)

**Fig. S1.** (A) Appearance of soybean plants during cultivation in greenhouse. (B) Salicylic acid application in a container. (C) Control soybean plants. (D) Soybean plants after SA application for 6h. (E) After 12 h. (F) After 24h. (G) After 36h. (H) After 48h.

(A) (B) (C)

(D) (E) (F)

****(G)

**Fig. S2.** Calibration curves of the isoflavones and coumestrol standard (A, daidzin; B, malonyldaidzin; C, daidzein; D, genistin; E, malonylgenistin; F, genistein; G, coumestrol).


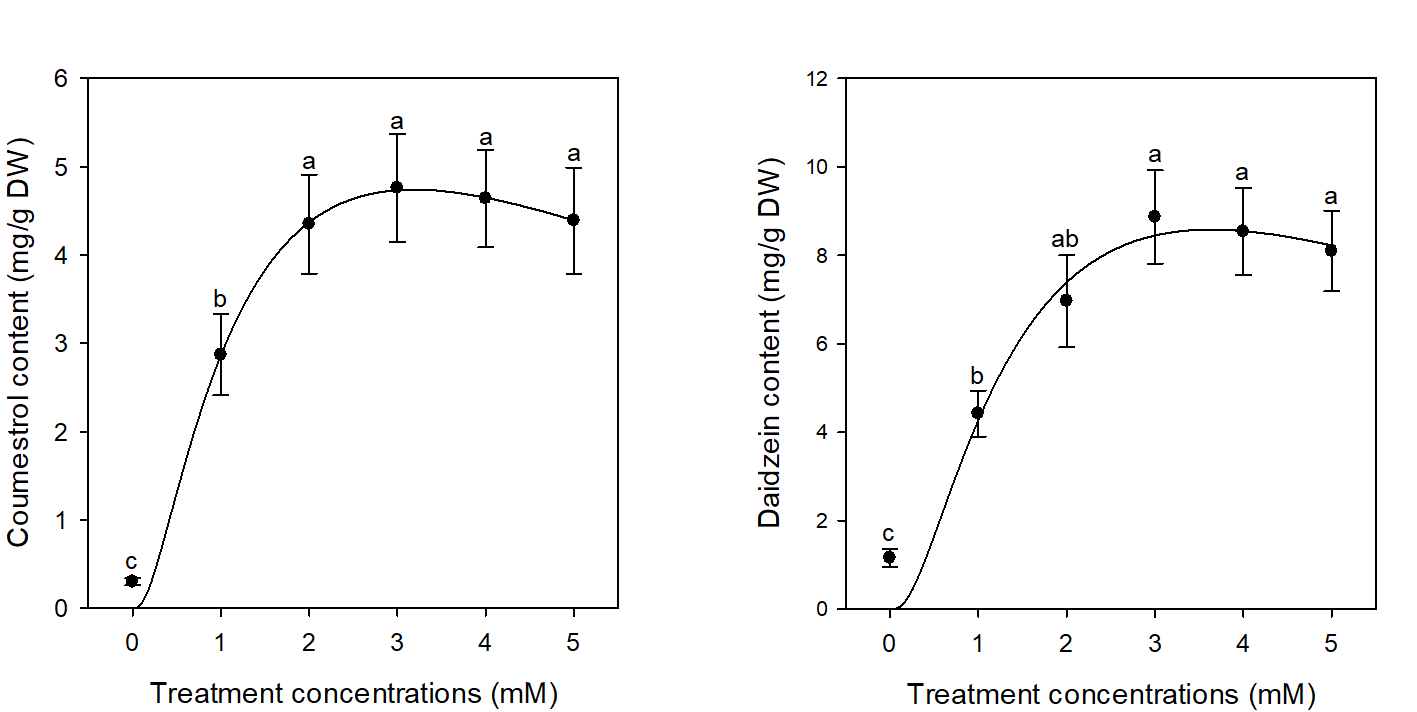
 (A) (B)

**Fig. S3.** (A) The changes in coumestrol level in soybean roots at different concentrations of salicylic acid treatment for 24 h. (B) The changes in daidzein level in soybean roots at different concentrations of salicylic acid treatment for 24 h. Different letters indicate significant differences, as determined by Tukey’s (LSD) test with *p* < 0.05.

(A)
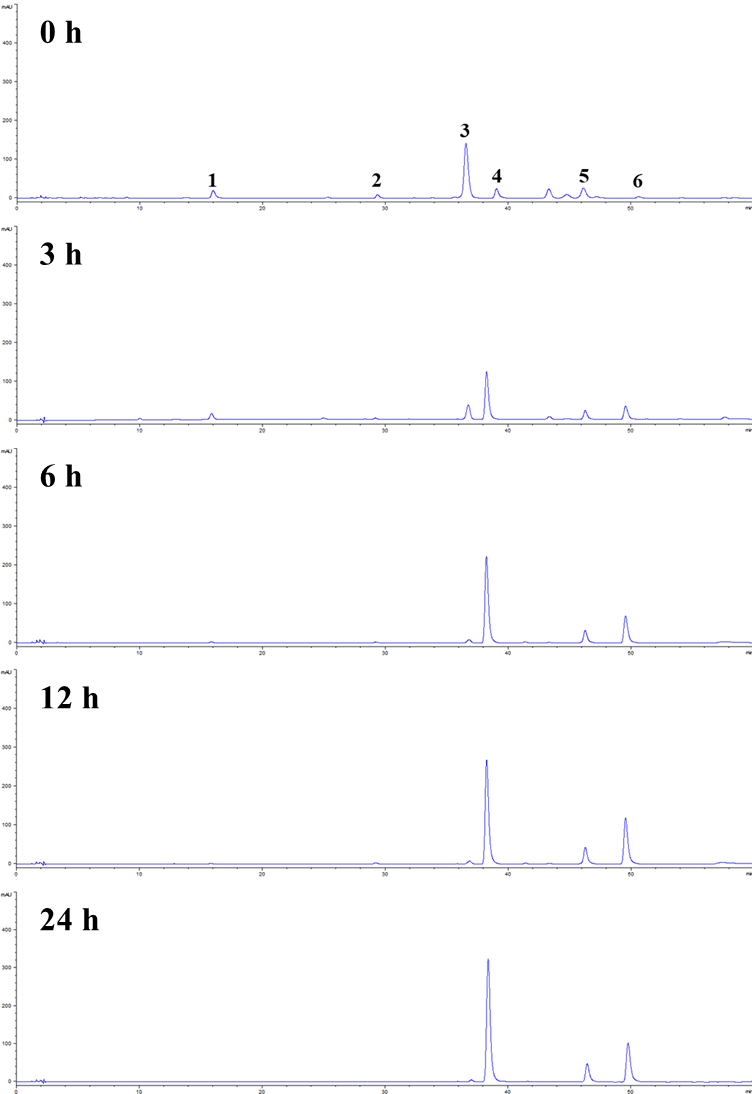


(B)

(C)

(D)

(E)

**Fig. S4.** The changes in HPLC chromatogram patterns of soybean roots at 254 nm UV during SA application. (A) 0h (control). (B) 3h after SA application. (C) After 6h. (D) After 12h. (E) After 24h. Peak 1, daidzin; peak 2, coumestrin; peak 3, malonyldaidzin; peak 4, daidzein; peak 5, malonylcoumestrin; peak 6, coumestrol.

* Most of phenolic compounds (**1**–**6**) were detected at 254 nm

(A)
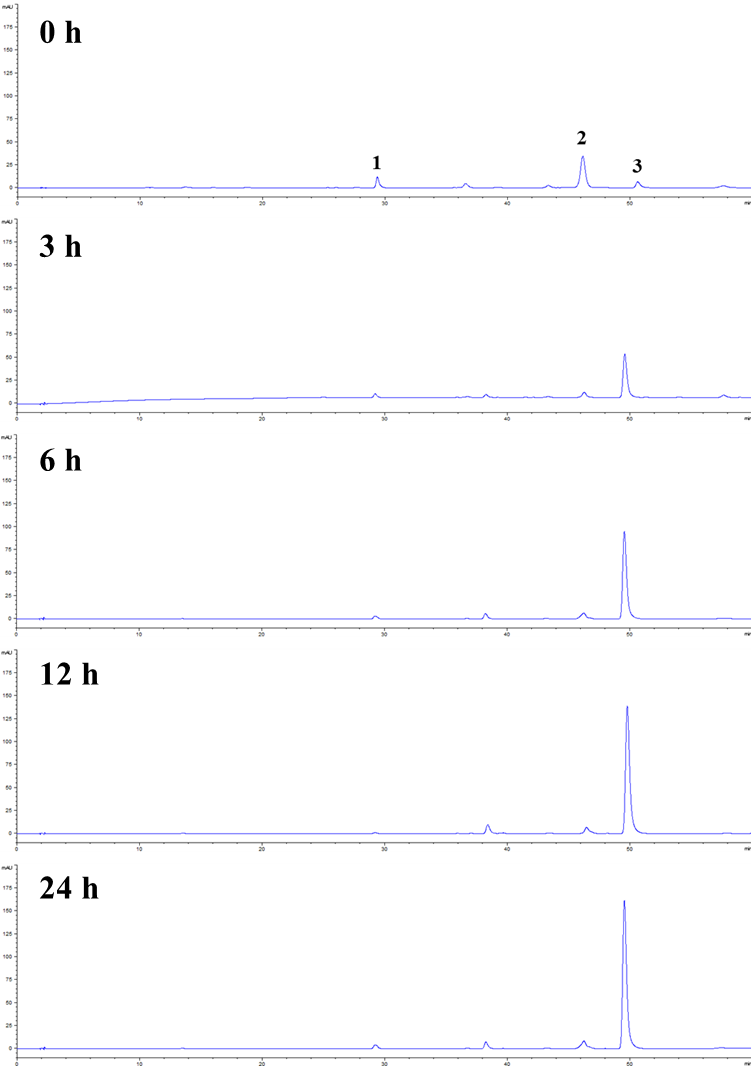


(B)

(C)

(D)

(E)

**Fig. S5** The changes in HPLC chromatogram patterns of soybean roots at 340 nm UV during SA application. (A) 0h (control). (B) 3h after SA application. (C) After 6h. (D) After 12h. (E) After 24h. Peak 1, coumestrin; peak 2, malonylcoumestrin; peak 3, coumestrol.

* Coumestrol (**3**) and its glycosides (**1** and **2**) were selectively detected at 340 nm.

**Quantification of coumestrol content in control soybean roots**

Coumestrin and malonylcoumestrin are not commercially available. Their quantification was carried out as coumestrol equivalent after hydrolysis by using β-glucosidase.

**Procedure of enzymatic hydrolysis**

For hydrolysis of coumestrol glycosides, 1 g of control soybean roots were extracted with 50 mL of 80% ethanol for 24 h at room temperature. The extract was evaporated to a volume of 10 mL, which was heated to 70 ºC to remove the malonyl group. The 1 mL of obtained extract was added to 4 mL of 50 mM phosphate buffer (pH 6.8) with 0.1 unit of β-glucosidase from almonds (Sigma-Aldrich Co., Ltd). The mixture was incubated at 37 °C for 2 h to hydrolyze the glucose moiety completely. The quantitative analysis was carried out by HPLC at 340 nm where only coumestrol derivatives can be detected selectively.

**Results**

**
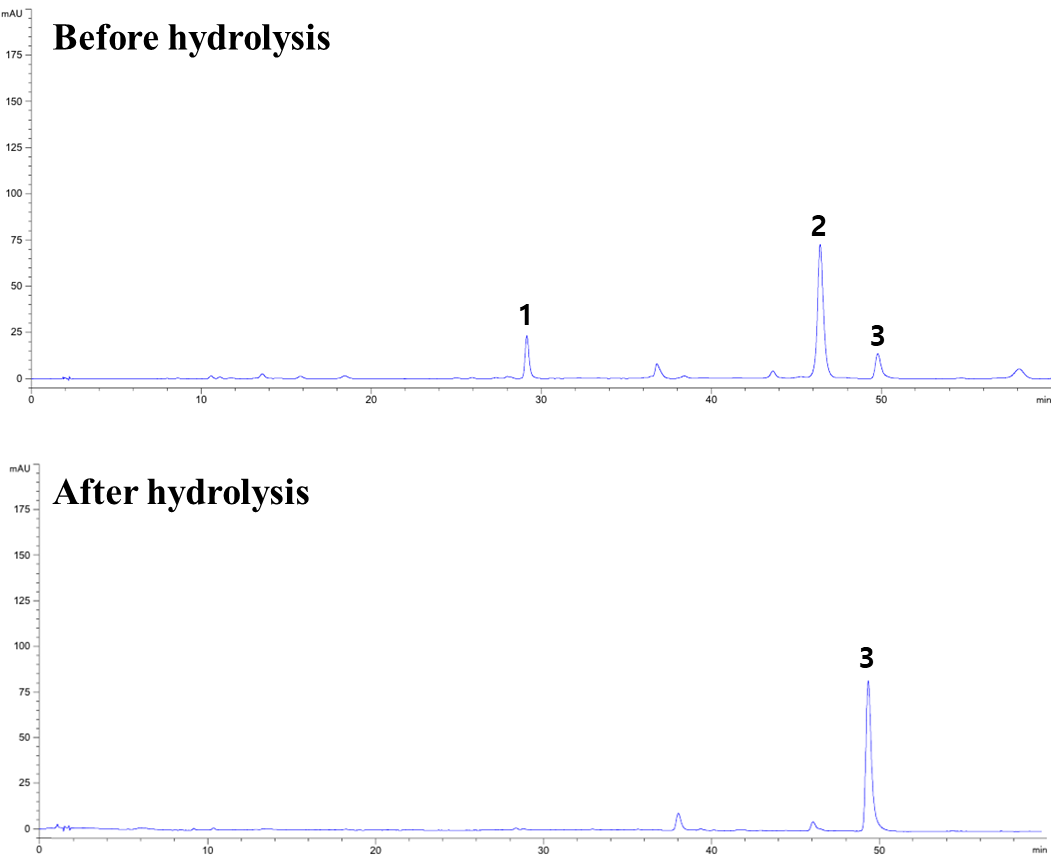
** **(A)**

**(B)**

**Fig. S6.** HPLC chromatograms observed at 340 nm UV. (A) HPLC chromatogram of control soybean roots extract. (B) HPLC chromatogram of hydrolyzed soybean roots extract. Peak 1, coumestrin; peak 2, malonylcoumestrin; peak 3, coumestrol.

**Summary**

Total coumestrol content in control soybean roots was estimated to be 2.14 mg/g DW after hydrolysis with β-glucosidase.

(A)
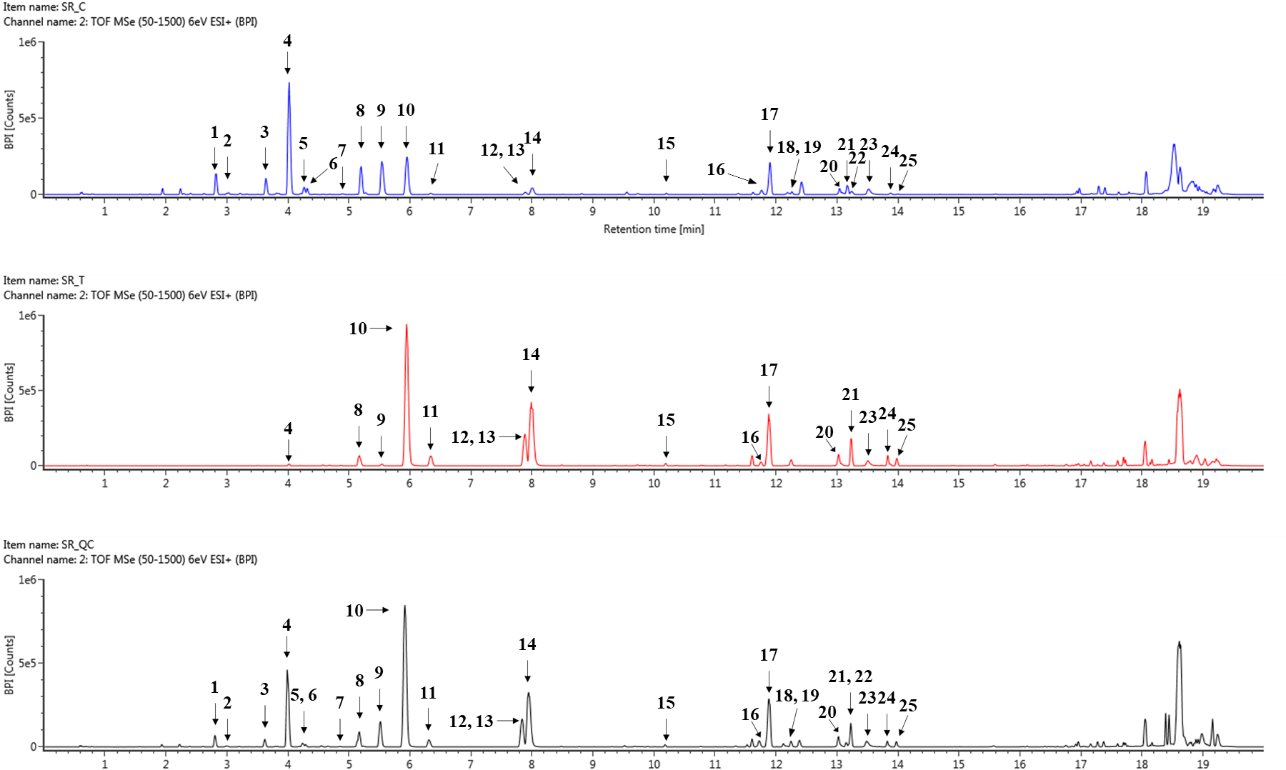


(B)


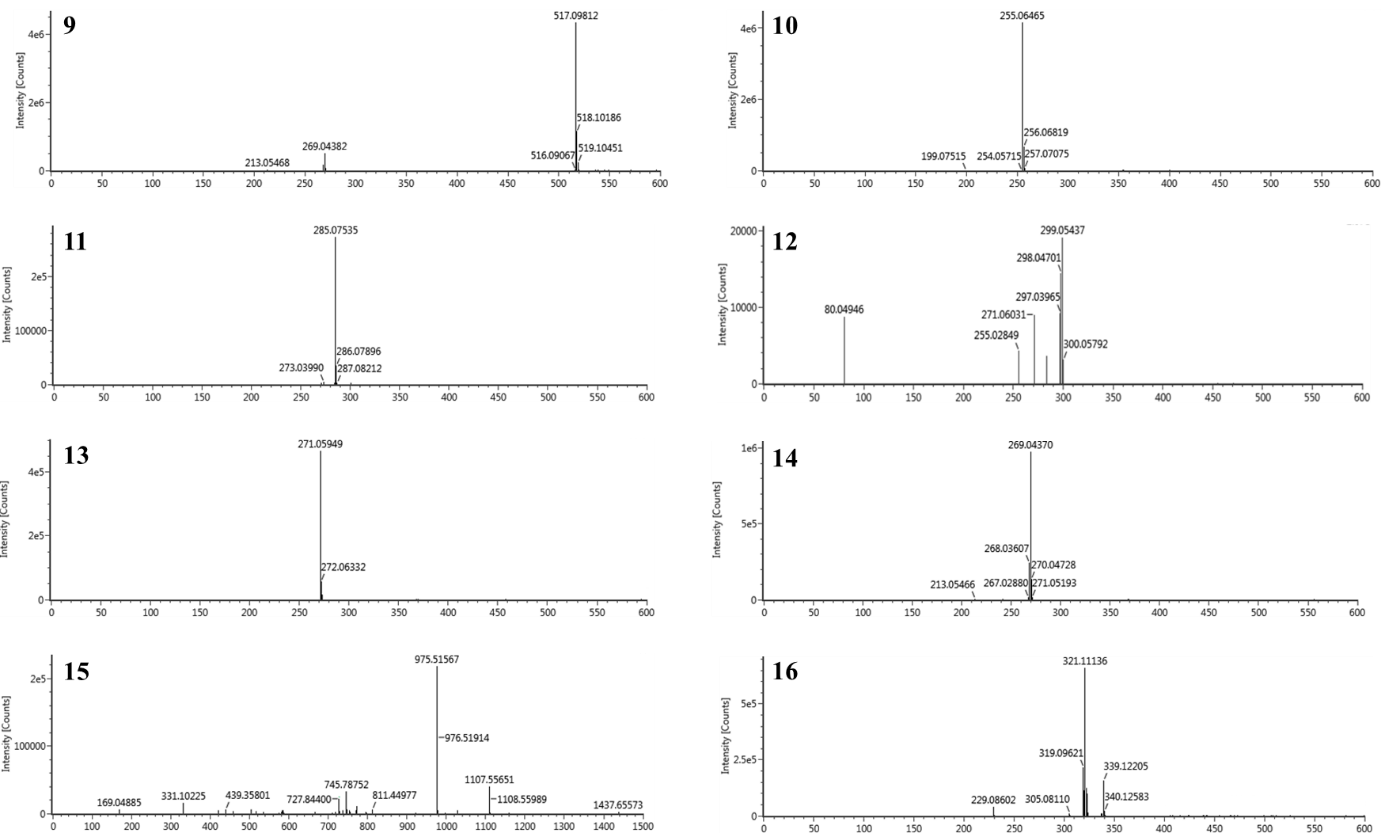

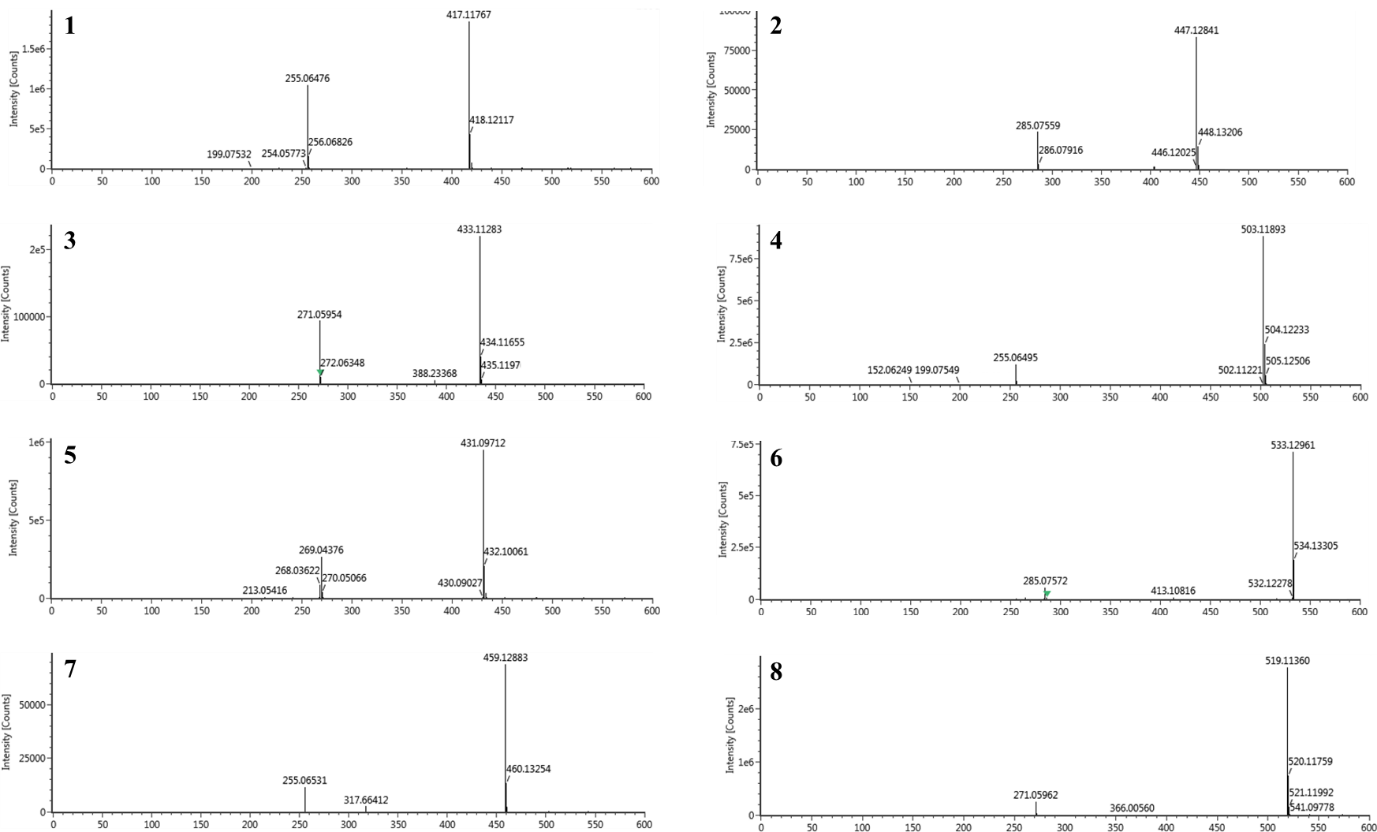
(C)


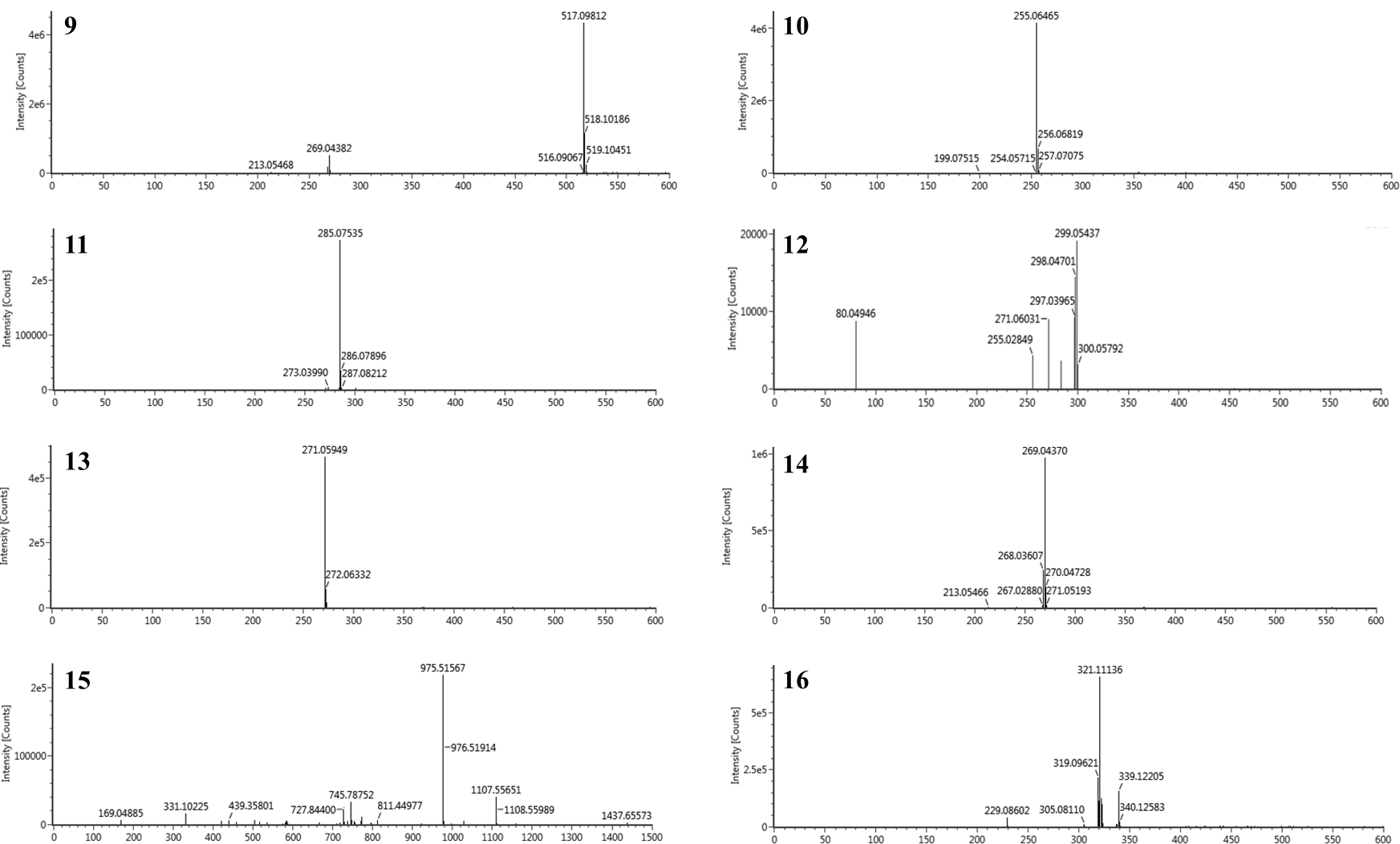


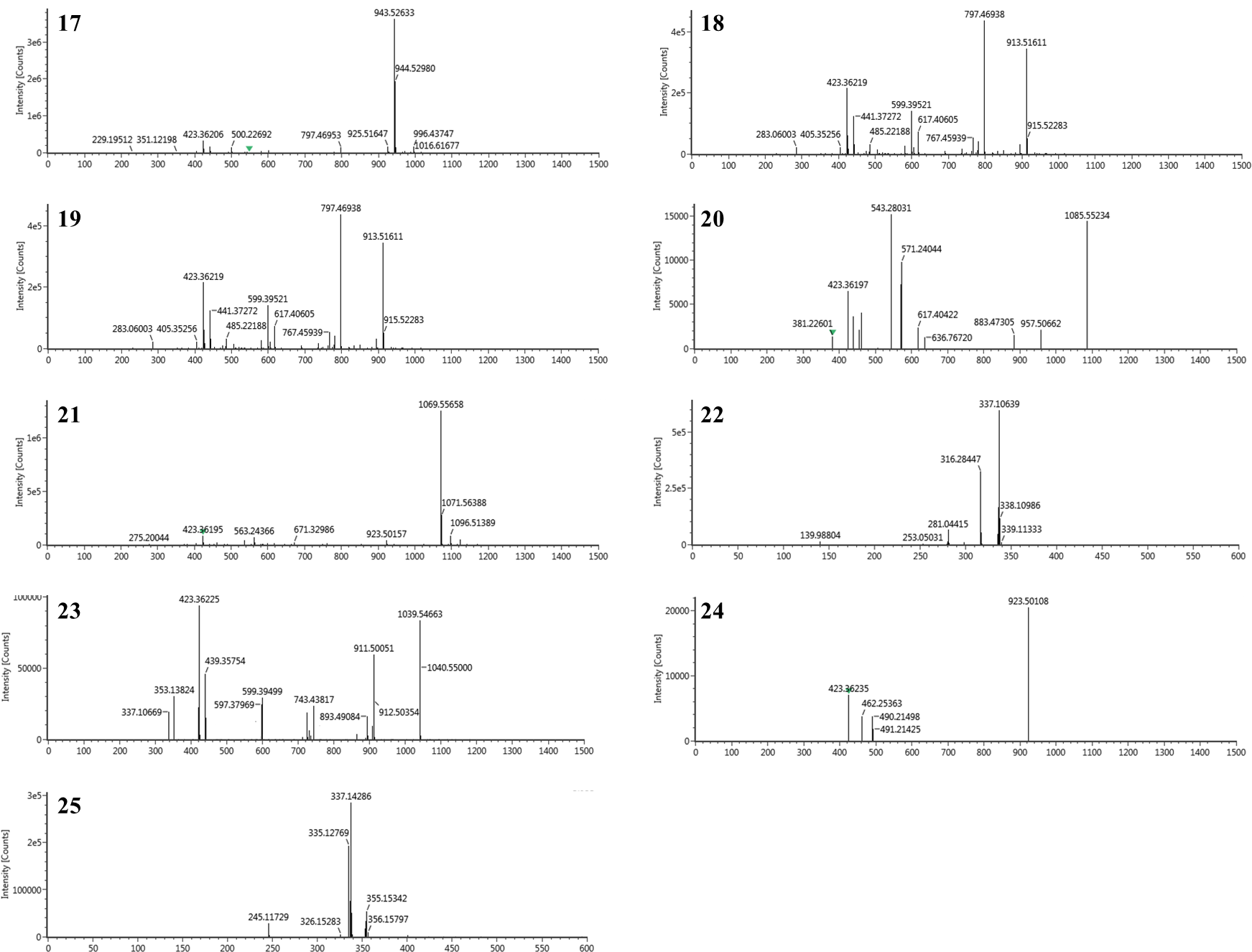


**Fig. S7.** (A) BPI chromatogram pattern of control soybean roots. (B) BPI chromatogram pattern of SA treated soybean roots. (C) Quality control (QC) by LC-TOF/MS. Mass fragmentation patterns of identified compounds in the soybean roots. 1, daidzin; 2, glycitin; 3, genistin; 4, malonyldaidzin; 5, coumestrin; 6, malonylglycitin; 7, acetyldaidzin; 8, malonylgenistin; 9, malonylcoumestrin; 10, daidzein; 11, glycitein; 12, isotrifoliol; 13, genistein; 14, coumestrol; 15, soyasaponin Ab; 16, glyceollin Ⅰ; 17, soyasaponin Ⅰ; 18, soyasaponin Ⅲ; 19, soyasaponin Ⅱ; 20, soyasaponin αg; 21, soyasaponin βg; 22, phaseol; 23, soyasaponin βα; 24, soyasaponin γg; 25, glyceollin Ⅳ.


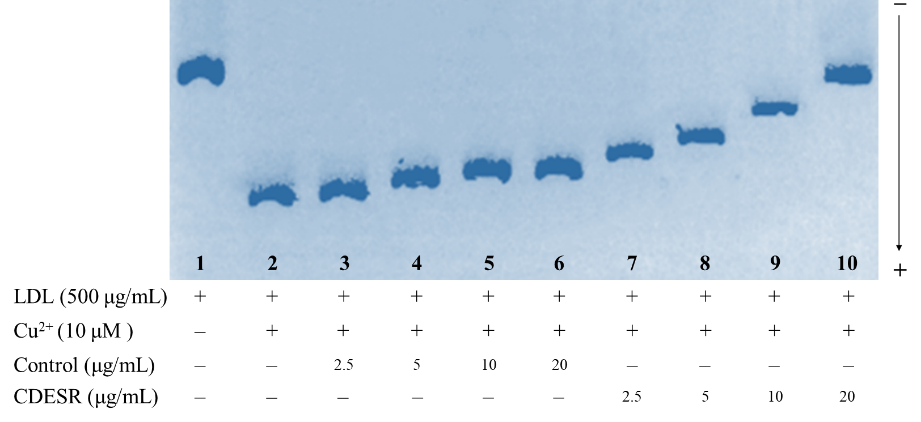


**Fig. S8.** Dose-dependent effects of control soybean roots and CDESR in REM assay. Lane 1, native LDL; lane 2, oxidized LDL; lane 3–6, control roots (2.5 ~ 20 μg/mL); lane 7–10, CDESR (2.5 ~ 20 μg/mL).

| Peak | *t*_R_ (min) | Compounds | Exact mass  [M+H]^+^ (*m*/*z*) | Fragment ions  [M+H]^+^ (*m*/*z*) | VIP | *p*-value |
| --- | --- | --- | --- | --- | --- | --- |
| 1 | 2.82 | Daidzin | 417.1176 | 255 | 1.27 | 3.92E-07 |
| 3 | 3.64 | Genistin | 433.1128 | 271 | 1.26 | 4.50E-07 |
| 4 | 4.02 | Malonyldaidzin | 503.1189 | 255 | 1.29 | 1.73E-08 |
| 5 | 4.27 | Coumestrin | 431.0971 | 269 | 1.25 | 1.36E-06 |
| 6 | 4.31 | Malonylglycitin | 533.1296 | 285 | 1.26 | 6.30E-07 |
| 8 | 5.20 | Malonylgenistin | 519.1136 | 271 | 1.30 | 5.18E-15 |
| 9 | 5.54 | Malonylcoumestrin | 517.0981 | 269 | 1.29 | 1.70E-08 |
| 10 | 5.95 | Daidzein | 255.0646 |  | 1.28 | 9.00E-09 |
| 11 | 6.34 | Glycitein | 285.0753 |  | 1.26 | 5.09E-07 |
| 12 | 7.86 | Isotrifoliol | 299.0543 | 271, 255 | 1.11 | 1.18E-05 |
| 13 | 7.89 | Genistein | 271.0595 |  | 1.29 | 1.04E-08 |
| 14 | 8.00 | Coumestrol | 269.0437 |  | 1.29 | 7.70E-09 |
| 15 | 10.20 | Soyasaponin Ab | 1437.6557 | 975, 439, 169 | 0.83 | 5.17E-03 |
| 17 | 11.90 | Soyasaponin Ⅰ | 943.5263 | 423 | 1.21 | 6.96E-08 |
| 18 | 12.26 | Soyasaponin Ⅲ | 797.4693 | 423 | 1.12 | 7.10E-06 |
| 19 | 12.26 | Soyasaponin Ⅱ | 913.5161 | 617, 441, 423 | 1.26 | 1.96E-10 |
| 21 | 13.17 | Soyasaponin βg | 1069.5565 | 563, 423 | 1.20 | 2.06E-05 |
| 23 | 13.46 | Soyasaponin βα | 1039.5466 | 743, 423 | 1.15 | 9.48E-05 |

**Table S1.** Identification of major metabolites contributing the separation among sample groups

**Table S2**. Effects of each compound or extract on oxidized LDL induced by Cu^2+^.

All samples were examined in triplicate. ^a^IC_50_ values of samples represent the concentration that caused 50% oxidation LDL by measurement of TBARS assay; ^b^Lag time of conjugated diene formation by oxLDL at 5 µg/mL of compounds or soybean root extracts; ^c^Probucol was used as a positive control.

| Sample | IC_50_^a^ (μg/mL) | Lag time^b^ (min) |
| --- | --- | --- |
| Coumestrol | 2.0 ± 0.3 | >240 |
| Daidzein | – | 90 |
| Control soy roots | 108.9 ± 1.9 | 115 |
| Treated soy roots | 36.1 ± 1.2 | 190 |
| Probucol^c^ | 21.9 ± 2.5 | 60 |
